# Digital mental health in Italy: findings from the multicentric DIGIT-PSY study

**DOI:** 10.3389/fpsyt.2025.1642455

**Published:** 2025-08-29

**Authors:** Laura Orsolini, Giulio Longo, Mario Luciano, Gaia Sampogna, Andrea Aguglia, Stefano Barlati, Giuseppe Blasi, Paola Calò, Claudia Carmassi, Giuseppe Carrà, Giovanni Castellini, Armando D’Agostino, Pasquale De Fazio, Chiara De Panfilis, Giorgio Di Lorenzo, Matteo Di Vincenzo, Carla Gramaglia, Valeria Latorre, Mirko Manchia, Giovanni Martinotti, Marco Menchetti, Mauro Pettorruso, Federica Pinna, Gabriele Sani, Gianluca Serafini, Maria Salvina Signorelli, Sarah Tosato, Antonio Ventriglio, Umberto Volpe, Andrea Fiorillo

**Affiliations:** ^1^ Unit of Clinical Psychiatry, Department of Clinical Neurosciences/Department of Experimental and Clinical Neurosciences (DIMSC), School of Medicine, Polytechnic University of Marche, Ancona, Italy; ^2^ Department of Psychiatry, University of Campania “L. Vanvitelli”, Naples, Italy; ^3^ Department of Neuroscience, Rehabilitation, Ophthalmology, Genetics, Maternal and Child Health (DINOGMI), Section of Psychiatry, University of Genoa, Genoa, Italy; ^4^ Istituto di Ricovero e Cura a Carattere Scientifico (IRCCS) Ospedale Policlinico San Martino, Genoa, Italy; ^5^ University of Brescia, Azienda Socio-Sanitaria Territoriale (ASST) Spedali Civili of Brescia, Brescia, Italy; ^6^ Psychiatric Neuroscience Group, Department of Translational Biomedicine and Neuroscience, University of Bari “Aldo Moro”, Bari, Italy; ^7^ Department of Mental Health, Azienda Sanitaria Integrata Giuliano-Isontina, Lecce, Italy; ^8^ Department of Clinical and Experimental Medicine, University of Pisa, Pisa, Italy; ^9^ Department of Medicine and Surgery, University of Milan Bicocca, Milan, Italy; ^10^ Psychiatry Unit, Department of Health Sciences, University of Florence, Florence, Italy; ^11^ Department of Health Sciences, University of Milan, Milan, Italy; ^12^ Psychiatric Unit, Department of Health Sciences, University Magna Graecia, Catanzaro, Italy; ^13^ Department of Medicine and Surgery, Unit of Neuroscience, University of Parma, Parma, Italy; ^14^ Department of Systems Medicine, University of Rome Tor Vergata, Rome, Italy; ^15^ Department of Translational Medicine, Università del Piemonte Orientale, Novara, Italy; ^16^ Psychiatry Division, Azienda Ospedaliero Universitaria Maggiore della Carità, Novara, Italy; ^17^ Complex Organization Unit Psychiatric Diagnosis and Care Service UO San Paolo, Azienda Sanitaria Locale (ASL) Bari, Bari, Italy; ^18^ Section of Psychiatry, Department of Medical Sciences and Public Health, University of Cagliari, Cagliari, Italy; ^19^ Department of Neuroscience, Imaging and Clinical Sciences, University “G. D’Annunzio” of Chieti-Pescara, Chieti, Italy; ^20^ Department of Biomedical and Neuromotor Sciences, University of Bologna, Bologna, Italy; ^21^ Department of Neuroscience, Section of Psychiatry, University Cattolica del Sacro Cuore, Rome, Italy; ^22^ Fondazione Policlinico A. Gemelli Istituto di Ricovero e Cura a Carattere Scientifico (IRCCS), Rome, Italy; ^23^ Department of Clinical and Experimental Medicine, Azienda Ospedaliero Universitaria (AOU) Policlinico Hospital, University of Catania, Catania, Italy; ^24^ Section of Psychiatry, Department of Neuroscience, Biomedicine and Movement Sciences, University of Verona, Verona, Italy; ^25^ Department of Clinical and Experimental Medicine, University of Foggia, Foggia, Italy

**Keywords:** digital literacy, digital mental health, digital psychiatry, digital readiness, mental health professionals, psychiatry

## Abstract

**Introduction:**

Despite an increasing interest in upscaling the digitalization process in mental health care services, there is still a gap in a deeper knowledge of the main barriers and facilitators allowing a capillary and consolidated implementation of digital mental health (DMH) and digital psychiatry (DP), particularly in the Italian context. A multicentric nationwide study (DIGIT-PSY) was designed with the aim to overview the current digitalization level of Italian mental health systems and professionals, by investigating needs/gaps to be addressed to accelerate the availability/access to DMH/DP interventions in Italy, as well as specific internal/external determinants of the process. The final goal of the DIGIT-PSY was to provide a roadmap for implementation strategies to reach a satisfactory level of digitalization of mental health care settings in Italy.

**Methods:**

A survey was distributed to a multiprofessional cohort of mental health professionals (psychiatrists, psychiatry trainees, psychologists, technicians in psychiatric rehabilitation and professional mental health educators), from public and private Italian settings, from May 1st, 2023 to September 30th, 2023. Internal/external determinants influencing the level of digitalization, as assessed by using the Digitalization index (DIGi score) were explored, by also comparing mental health (MH) professionals in three groups: a) low (DIGi ranged 4-9); b) moderate (DIGi ranged 10-15); and, c) high level of digitalization (DIGi ranged 16-20).

**Results:**

Only 16.4% of the sample declared an optimal/good clinical practice experience in the field of DP/DMH interventions, being mostly among psychiatrists and psychologists and those who currently practice psychotherapy (all, p < 0.001). Only 6.5% (N = 90) of mental health professionals received a formal DMH/DP training. The mean DIGi index was 9.9 ± 3.5. MH professionals owning a pre-COVID-19 and/or post-COVID-19 clinical experience in DMH/DP showed the highest DIGI scores (both p<0.001), suggesting a COVID-19 effect in boosting the digitalization of MH services. Working with DMH/DP knowledgeable colleagues and with colleagues who routinely deliver DMH/DP in their clinical practice increased the digitalization level of MH professionals and services (both p<0.001). Both education/training in DMH/DP (p=0.002) and regular clinical practice in DMH/DP (p<0.001) improved the chance to reach a high DIGi score. While working in a public job setting negatively predicted the DIGi index (p=0.007).

**Discussion:**

National initiatives should firstly address education and training needs of the youngest mental health professionals, particularly those without a mentor/supervisor experienced in providing DMH/DP and in educating younger professionals in the digital clinical practice. Sex-sensitive consolidating strategies should be implemented in those mental health services already digitalized. Longitudinal studies should evaluate the efficacy in the long-term of country-based digitalization strategies.

## 1 Introduction

The COVID-19 pandemic significantly accelerated the use of digital mental health (DMH) and digital psychiatry (DP), particularly telepsychiatry (TP), within mental health services worldwide (1–3). Several studies demonstrated that DMH/DP are fully comparable to the face-to-face interventions in different target populations and settings, in terms of efficacy and effectiveness (4–8). Within this context, there is an increasing interest in upscaling the digitalization process in different healthcare settings, including mental health services, as underlined by the World Health Organization (WHO) global initiative on digital health (9) and more recently also supported by the European Psychiatry Association (EPA) (10) and the World Psychiatry Association (WPA) Task Force on Digital Psychiatry (11–14). However, despite these international global initiatives, there is still a gap in a deeper knowledge and understanding of which determinants in terms of barriers and facilitators might influence the beginning and consolidating process of the digitalization of mental health care, particularly in favoring a long-term and sustainable implementation of DMH/DP interventions in all real-world mental health care settings (15, 16). To date, several individual, social and structural determinants have been proposed, including the level of acceptability, feasibility and sustainability in providing DMH/DP by mental health professionals and their mental health services and infrastructures; the level of digital/technological literacy (knowledge and/or expertise) by patients and/or mental health professionals; the level of education, training and experience on DMH/DP interventions, also in the different mental health disorders (1, 3, 11, 17–20).

At the time of writing of the current study, no research has been published specifically targeting the determinants of the digitalization process of mental health care in the Italian context. Hence, a multicentric Italian nationwide study (DIGIT-PSY study) was designed with the aim to primarily overview the current digitalization level of Italian mental health systems and mental health professionals, by recruiting a cohort consisting of physicians (trained in psychiatry and psychiatry trainees), psychologists and mental health professionals working in the field of psychiatric rehabilitation and psychosocial interventions (technicians in psychiatric rehabilitation [PRT] and professional mental health educators [PE]). The sample was collected among both public (not-university and university inpatient and territorial outpatient services) and private (outpatient services and clinics) settings.

### 1.1 Aims

- Given the lack of Italian studies investigating the current level of digitalization of mental health services and mental health professionals, the primary aim was to investigate which is the current situation in Italy in terms of knowledge and digital proneness of mental health services and professionals, supposing regional differences in terms of mental health services (depending on financial resources) and differences across all mental health professionals (depending on the type of working setting and/or type of training/education level). These findings could be helpful to identify which are the current needs and gaps that should be addressed in Italy to increase the availability and access to DMH/DP interventions in mental health services and infrastructures, from the clinicians’ perspective.
- Secondary aims included:
  - Evaluating if any internal (e.g., socio-demographic variables, belonging generation, professional variables such as number of clinical experience, type of profession, type of job settings, etc.) could act as factors influencing the digitalization process within mental health care in the Italian context, by hypothesizing sex- and age-based differences and supposing a higher level of digitalization proneness in academic contexts (mainly due to the supposed higher chance to be trained in DMH/DP);
  - Evaluating if any external (e.g., associated with the predominant working setting, regional differences, and so forth) determinants could act as factors influencing the digitalization process within mental health care in the Italian context, by hypothesizing regional differences depending on financial resources and the different impacting role of the accelerated digitalization due to the COVID-19 pandemic. In particular, one hypothesis was to find higher levels of digitalization of mental health services and professionals in Northern Italian regions compared to the Southern and Central ones (mainly due to the different COVID-19 wave effect in accelerating the implementation of digitalization);
- The final goal of the DIGIT-PSY was to provide a roadmap for implementation strategies to reach a satisfactory level of digitalization of mental health care settings in Italy, by hypothesizing the need to tailor the strategies considering the regional differences, the type of mental health professional, the differential influence of the abovementioned internal and/or external factors.

## 2 Methods

### 2.1 Study design and sample recruitment strategy

An Italian multicentric no-profit observational naturalistic cross-sectional study was carried out through an *ad hoc* developed questionnaire that was administered hand-by-hand and online by using the European platform EUSurvey^®^ to a cohort of mental health professionals recruited by 27 Italian University collaborating centers in the timeframe from May 1, 2023 to September 30, 2023. All mental health professionals who met the following inclusion criteria have been included in the analysis of our study: a) subjects belonging to the following professional categories: M.D. specialized in psychiatry, M.D. psychiatry trainees, psychologists with or without specialization in psychotherapy, PRT and PE; b) all subjects who agreed to participate in the study; c) having provided a written informed consent; d) all subjects who authorized the treatment of sensible and personal data for research purposes. While all subjects who disagree to participate in the study or who did not fill out all sections of the survey have been removed by the dataset.

Sample size was calculated using the Statistical Software G*Power version 3.1. (Franz, Universitat Kiel, Germany), by keeping the values of confidence level as 99%, anticipated population proportion 0.5, an α error of 0.05, a power of 95%, considering the primary outcome and taking into consideration all variables to be entered in the multivariable analysis, in order to obtain at least an effect size of >0.3. A total sample size of 1,302 mental health professionals was established to be reached for the present study, composed of at least 325 M.D. specialized in psychiatry, 325 M.D. psychiatry trainees, 325 psychologists, 325 mental health professionals working in psychiatric rehabilitation (PRT and PE). For each collaborating center was established a threshold of at least 13 participants to be recruited for each of the abovementioned categories to be included in the following phases of the study.

Participation was voluntary and only processed once a written informed consent was obtained. The study was conducted in accordance with the ethical principles outlined in the Declaration of Helsinki and according to the guidelines for Good Clinical Practice (GCP) (9) and according to the CHERRIES guidelines (21), following the approval by the Ethical Committee of the Marche Region (protocol code DIGIT-PSY n. 62/2023).

### 2.2 The structure of the survey

A preliminary questionnaire was initially developed after a literature search carried out by L.O. and U.V. about all currently available and used tools assessing a set of indices/variables related to DMH (i.e., digital literacy, digital readiness, acceptability, social influence, feasibility, satisfaction and feasibility/access). After three virtual meetings performed by consulting a small focus group of Italian experts belonging to the Digital Mental Health working group of the Italian Society of Social Psychiatry (SIPS) (U.V., L.O., G.C., A.V., M.S.S., G.D.L., P.C. and G.S.), the definitive draft of the questionnaire (**Supplementary File 1**) was developed and approved to be circulated. The final questionnaire was constituted by six sections.

In the current paper, it was analyzed specifically the first and second sections of the questionnaire. The first section included a set of socio-demographic and professional/job variables structured in 9 items (of which 5 multiple choice answer questions, and 4 open-ended questions). The second section investigated the level of mental health professionals’ clinical experience in delivering DP/DMH interventions in their clinical practice, also investigating the level of clinicians’ theoretical and/or practical expertise and training on DMH/DP and the level of digitalization of mental health professionals’ professionals’ service/infrastructure. The second section was developed by consulting studies carried out by Dorés et al. (22) and Sander et al. (23). The second section comprise 13 questions, of which 1 item with a dichotomous answer yes/no (item 1) regarding previous experience in delivering DMH/DP interventions in the pre-COVID-19 era; and 12 questions with a 5-Likert scale answer (from “1 = never” to “5 = always”) investigating the level of knowledge and use of DMH by the mental health professional, his/her colleagues and his/her mental health service as well as his/her propensity to recommend a DMH/DP intervention to his/her patients. Item 2 was also transformed into a dummy variable (presence versus absence). The DIGi (Digitalization) index was developed to assess the extent to which mental health professionals and systems support the digitalization process through structured clinical practices and internal policies. The score is based on a set of items (item 2, 4, 5 and 6) derived by the consultation of studies carried out by Dorés et al. (22) and Sander et al. (23). To ensure content validity, the items were reviewed by a panel of experts belonging to the Digital Mental Health working group of the Italian Society of Social Psychiatry (SIPS), as described above. The instrument has been piloted with a sample representative of the target population, and item performance was analyzed to refine the index. Internal consistency of the DIGi index was evaluated using Chronbach’s alpha which yielded a value of 0.84, indicating a good reliability. The DIGi score was used to stratify mental health professionals in the following three groups: a) low level of digitalization (DIGi ranged 4-9); b) moderate level of digitalization (DIGi ranged 10-15); c) high level of digitalization (DIGi ranged 16-20).

### 2.3 Statistical analyses

Statistical analyses were carried out by using the Software Statistical Package for Social Sciences (SPSS) for MacOS (version 26.0, IBM Corp., Armonk NY). The significance level was set *a priori* at p ≤ 0.05, and all hypotheses were two-tailed. All categorical variables were summarized as absolute frequencies (n) and percentages (%), while all continuous variables have been summarized as means (m) and standard deviations (SD) or median (M) and 95% Confidence Interval (CI), based on the normal or non-normal distribution. The normality of the DIGi index (as continuous variable) and all other quantitative variables were verified by using the Kolmogorov-Smirnov and Shapiro-Wilk normality tests. The variables ‘age’ and ‘number of years of clinical experience’ were logarithmically transformed in order to obtain a normally distributed variable (respectively, ln_age and ln_clinical_experience_years). Participants were firstly stratified in four groups according to the professional category (M.D. specialized in psychiatry, M.D. psychiatry trainees, psychologists and technicians [PRT and PE]). The χ2 test and Fisher-Freeman-Halton’s test were used to compare all categorical variables across all four professional categories and across all three Italian geographical areas (Northern, Central and Southern regions). The Analysis of Variance (ANOVA) or Kruskall-Wallis test were performed to compare all quantitative variables across all four professional categories and across all three Italian geographical areas (Northern, Central and Southern regions), depending on the normality distribution of qualitative variables.

Furthermore, participants were stratified based on the digitalization index (DIGi) in three groups: “high” (ranged 16-20), “moderate” (ranged 10-15) and “low” level of digitalization (ranged 4-9). The Analysis of Variance (ANOVA) or Kruskall-Wallis test, where appropriate, were used to perform all comparisons between quantitative variables across three groups according to the DIGi index. Three DIGi groups will be compared by using also Pearson’ χ² test regarding all socio-demographic and structural/environmental features of mental health services/infrastructures and other categorical variables, such as the current professional/academic role, type of job settings, job region/zone, presence/absence of a pre-COVID-19 previous experience in DP/DMH, presence/absence of a theoretical and/or practical training in DP/DMH, and so forth). The DIGi index will be also used as a continuous variable. Independent student’s T-test or two-tailed Mann-Whitney’s U test, where appropriate, were performed to compare DIGi index according to the following dichotomous variables: sex, type of job setting (public versus private), presence/absence of a pre-COVID-19 previous experience in DP/DMH, presence/absence of a previous experience in DP/DMH in the last 3 years, having colleagues who know and/or deliver DMH/DP interventions in my own workplace, presence/absence of DMH/DP training, knowledge and experience, independently by the COVID-19 outbreak. The Analysis of Variance (ANOVA) was run to perform all comparisons of the DIGi index according to the following variables: four professional categories and three geographical areas. The DIGi index (as a quantitative variable) has been also entered within a stepwise multivariate linear regression model in order to explore which independent internal and/or external determinants could act as predictor of the level of digitalization.

## 3 Results

The survey was filled out by 1,402 respondents, of which 13 were excluded in the analysis due to their refusal to participate in the study. A total number of questionnaires correctly fulfilled during the collection process and afterwards included in the downstream analysis was 1,389.

### 3.1 Sample characteristics

The final sample included 1,389 participants, of which 987 males (71.1%). The mean age of participants is 37.0 (± SD = 10.0) years, with an average ln_age mean of 3.6 (± SD = 0.3). The median number of years of clinical experience is 8.0 (CI% 10.2-11.2) ranging from less than 1 year to 50 years, with an average ln_clinical_experience_years mean of 1.9 (± SD = 1.0). Among mental health professionals, 340 participants are M.D. specialized in Psychiatry, 377 M.D. psychiatry trainees, 328 psychologists (of which 248 with a certified psychotherapy training), and 344 mental health professionals working in psychiatric rehabilitation (of which 251 PRT, 72.9%). Regarding job setting, most participants work in public settings (N = 962; 69.3%), most of them in public outpatient services (N = 401; 41.7%) and in university hospitals (N = 381; 39.6%). Within the sample of physicians and psychologists, around 50.2% of them (N = 525) declared to have been formally trained in psychotherapy, mostly among psychologists (N = 304; 92.7%), followed by psychiatrists (N = 162; 47.6%). Most psychotherapists were trained in cognitive-behavioral psychotherapy (CBT) (N = 275; 52.4%), followed by psychodynamic approaches (N = 162; 30.9%). The sample was enough equally distributed across different Italian regions, comprising mental health professionals mainly working in the Southern regions (N = 526; 37.9%), followed by Central Italy (N = 489; 35.2%) and the Northern Regions (N = 374; 26.9%) (**Table 1**). The geographical areas are homogeneously distributed by sex (p = 0.193), while mental health professionals from central regions significantly displayed a higher age compared to other two geographical areas (p < 0.001) and a lower number of years of clinical experience compared to those coming from southern regions (p = 0.002).

**Table 1:** Socio-demographic characteristics of the sample, across all four professional categories.

	Total sample (N = 1,389)	M.D. Psychiatrists (N = 340)	M.D. Psychiatry trainees (N = 377)	Psychologists (N = 328)	Mental health professionals working in psychiatric education and/or rehabilitation (N = 344)
Sex					
Males, N (%)	987 (71.1%)	194 (57.1%)	216 (57.3%)	286 (87.2%)	291 (84.6%)
Females, N (%)	402 (28.9%)	146 (42.9%)	161 (42.7%)	42 (12.8%)	53 (15.4%)
Geographical zone					
Northern Italy	374 (26.9%)	85 (25%)	111 (29.4%)	84 (25.6%)	94 (27.3%)
Central Italy	489 (35.2%)	125 (36.8%)	159 (42.2%)	112 (34.1%)	93 (27.0%)
Southern Italy	526 (37.9%)	130 (38.2%)	107 (28.4%)	132 (40.2%)	157 (45.6%)
Job setting					
Public, N (%)	1,032 (74.3%)	303 (29.4%)	376 (36.4%)	135 (13.1%)	218 (21.1%)
Private, N (%)	357 (25.7%)	37 (10.4%)	1 (0.3%)	193 (54.1%)	126 (35.3%)
Psychotherapy training					
none, N (%)	864 (62.2%)	178 (20.6%)	320 (37.0%)	24 (2.8%)	342 (39.6%)
yes, no current practice, N (%)	81 (5.8%)	43 (53.1%)	19 (23.5%)	18 (22.2%)	1 (1.2%)
yes, current practice, N (%)	444 (32.0%)	119 (26.8%)	38 (8.6%)	286 (64.4%)	1 (0.2%)
Age, mean (SD)	37.0 (10.0)	43.2 (9.8)	30.8 (4.8)	40.6 (9.6)	34.4 (10.1)
Clinical experiences, years, median (95%CI)	8.0 (10-2-11.2)	10.0 (11.3-13.3)	4.0 (6.8-8.6)	10.0 (10.6-12.6)	9.5 (10.7-12.7)

Northern Italy: Liguria, Lombardia, Piemonte, Trentino, Veneto; Central Italy: Abruzzo, Emilia Romagna, Lazio, Marche, Toscana; Southern Italy: Calabria, Campania, Puglia, Sardegna, Sicilia. N, sample; %, percentage; M.D., medical doctor; M, mean; SD, standard deviation; CI, Confidence Interval.

N, frequency; %: percentage; CI, Confidence Interval; M.D., medical doctor; SD, standard deviation.

### 3.2 Level of clinical experience in the field of DMH/DP

Most mental health professionals did not have previous clinical experience in delivering DMH/DP interventions before the COVID-19 pandemic (N = 1,209; 87%), without differences across sexes (p = 0.491) either across three geographical areas (p = 0.064) (**Table 2**). Statistically significant lower levels were reported among younger (p < 0.001), those clinicians with a lower number of years of clinical experience in mental health and care (p = 0.004) and mental health professionals working in public settings (p < 0.001). Among four professional categories, psychiatry trainees, PRT/PE significantly displayed a lower pre-COVID-19 clinical experience in delivering DMH/DP, compared to other professionals (all with p < 0.001).

**Table 2:** Clinical experience in digital mental health and digital psychiatry, across all professional categories.

	Total sample (N = 1,389)	M.D. Psychiatrists (N = 340)	M.D. Psychiatry trainees (N = 377)	Psychologists (N = 328)	Mental health professionals working in psychiatric education and/or rehabilitation (N = 344)	P-value*
pre-COVID19 DP/DMH experience, yes, N (%)	180 (13.0%)	72 (21.2%)	9 (2.4%)	67 (20.4%)	32 (9.3%)	χ2 (3) = 89.014; **p < 0.001**
Last 3 years DP/DMH experience, yes, N (%)	396 (28.5%)	127 (37.4%)	46 (12.2%)	151 (46%)	72 (20.9%)	χ2 (3) = 125.282; **p < 0.001**
DP/DMH recommended to own patients, yes, N (%)	222 (16%)	68 (30.6%)	28 (12.6%)	91 (41.0%)	35 (15.8%)	χ2 (3) = 67.067; **p < 0.001**
DP/DMH good knowledge, yes, N (%)	268 (19.3%)	92 (34.3%)	39 (14.6%)	93 (34.7%)	44 (16.4%)	χ2 (3) =59.186; **p < 0.001**
DP/DMH good experience, yes, N (%)	228 (16.4%)	81 (35.5%)	28 (12.3%)	90 (39.5%)	29 (12.7%)	χ2 (3) =80.837; **p < 0.001**
DP/DMH good training, yes, N (%)	90 (6.5%)	26 (28.9%)	15 (16.7%)	27 (8.2%)	22 (24.4%)	χ2 (3) =6.321; p = 0.097
Face-to-face interventions, yes, N (%)	1288 (92.7%)	317 (24.6%)	349 (27.1%)	314 (24.4%)	308 (23.9%)	χ2 (3) =9.734; **p = 0.021**

*Fisher-Freeman-Halton’s test.All significant p-values are provided in bold.

Most mental health professionals declared that they did not have any previous clinical experience in the field of DMH/DP during the last triennium (2020-2023) (N = 993; 71.5%) (**Table 2**). Statistically significant lower levels were reported among female mental health professionals (p = 0.002), younger (p < 0.001) and less experienced (p < 0.001) and those mental health professionals working in public settings (p < 0.001). Among four professional categories, psychiatry trainees, PRT/PE significantly displayed lower clinical experiences in delivering DMH/DP during the last triennium (2020-2023) (all with p < 0.001). Mental health professionals working in the Southern Italian regions significantly displayed lower clinical experience in delivering DMH/DP interventions, compared to Northern and Central areas (p = 0.040). Among those participants who provided an answer to the optional question regarding the main motivation underpinned the choice in not delivering DMH/DP interventions, most mental health professionals reported “I did not receive an enough training to apply DMH/DP interventions in my clinical practice” (35.5%; N = 327) followed by “I do not know how to use DMH/DP interventions in my clinical practice” (27.5%; N = 253) (**Table 2**).

Only 22.8% (N = 316) of the sample declared that DMH/DP interventions are well known by their working colleagues, mostly by mental health professionals who work in private settings (p < 0.001). No geographical differences were observed (p = 0.358). However, most participants reported that DMH/DP interventions are not commonly used by their colleagues (84.4%; N = 1,173), mainly by mental health professionals who work in public settings (p < 0.001) and those who declared to have colleagues with a limited/poor/absent knowledge about DP/DMH interventions (p < 0.001). No significant geographical differences were observed (p = 0.984).

Only 16% (N = 222) of mental health professionals reported that they “often” or “always” proposed a DMH/DP intervention to their patients, mainly among the categories of psychiatrists and psychologists (**Table 2**), older mental health professionals (p < 0.001) and those with longer clinical experience (p < 0.001), those working in private settings (p < 0.001) and those who currently practice psychotherapy (p < 0.001) without any statistically significant difference depending on the type of psychotherapy (p = 0.084). No significant differences based on sex (p = 0.778) or geographical area (p = 0.145) were found. While 28.6% (n=397) of mental health professionals declared to propose DP/DMH interventions “sometimes” to their own patients.

Only 19.3% (n=268) of recruited mental health professionals reported enough knowledge in the field of DMH/DP interventions, particularly among psychiatrists and psychologists (**Table 2**), while 29.9% (n=416) declared a limited knowledge. Those mental health professionals more knowledgeable were statistically significantly represented by those older (p < 0.001), with a longer clinical experience (p = 0.003), working in private settings (χ2 = 8.850; p = 0.003), those who currently practice psychotherapy (χ2 = 42.655; p < 0.001), without any statistically significant difference depending on the type of psychotherapy (χ2 = 1.570; p = 0.456). No significant differences based on sex (p = 0.335) or geographical area (p = 0.118) were observed.

Similarly, only 16.4% of the sample declared an optimal/good clinical practice experience in the field of DP/DMH interventions, being mostly among psychiatrists and psychologists and those who currently practice psychotherapy (all with p < 0.001), without any differences depending on the type of psychotherapy approach (p = 0.155) (**Table 2**). Among these, the oldest mental health professionals (p < 0.001), those with a longer clinical experience (p = 0.010) and working in private settings (p = 0.017) are those who declared the better clinical experience in delivering DMH/DP interventions. No significant differences were found based on the sex (p = 0.339) or geographical area (p = 0.852).

Only 6.5% (N = 90) mental health professionals declared to have previously received adequate training in the field of DMH/DP. Conversely, 14.1% (N = 196) of the sample reported poor/limited formal training in DMH/DP. No statistically significant differences were found based on the sex (p = 0.153), age (p = 0.051), number of years of clinical experience (p = 0.547), type of job setting (p = 0.061), professional category (p = 0.097), psychotherapy practice (p = 0.343) or type of psychotherapy approach (p = 0.927) or geographical area (p = 0.138).

Most professionals (92.7%; N = 1,288) declared that they much more likely recommend face-to-face traditional interventions rather than DMH/DP to their own patients. This pattern seemed to be much more likely reported by male professionals (p = 0.007). While those mental health professionals without a previous DP/DMH experience during the last triennium (2020-2023) and those without any DP/DMH clinical experience at all, are those who much more likely declared to prefer face-to-face modality compared to the digital modality (respectively, p = 0.003 and p = 0.017). No statistically significant differences were found based on the age (p = 0.259), number of years of clinical experience (p = 0.770), the type of job setting (p = 0.643), psychotherapy practice (p = 0.265), type of psychotherapy (p = 0.619), geographical area (p = 0.922), having a previous DP/DMH experience during the COVID-19 pandemic (p = 0.978), neither by the level of DP/DMH knowledge or practice among their own colleagues (respectively, p = 0.085 and p = 0.440). Whereas some professionals are much more prone to offer DMH/DP interventions to their patients, most of them reported delivering DMH/DP interventions only together with synchronous support by a therapist (31.2%; N = 433).

Interestingly, when asked about their general interest in providing DMH/DP interventions in the case their mental health service/infrastructure would be able to implement this therapeutic opportunity, only 38.1% (N = 530) of the sample declared a positive interest/predisposition while around 34.8% (N = 483) of them declared an “ambivalent” predisposition. No significant differences were found based on sex (p = 0.242), age (p = 0.187), number of years of clinical experience (p = 0.416), type of profession (p = 0.078), job setting (p = 0.106), psychotherapy practice (p = 0.727), type of psychotherapy (p = 0.455). Interestingly, among those much more likely ‘interested’ mental health professionals, most of them are significantly represented by those working with colleagues without DP/DMH clinical experience/practice (p < 0.001) or knowledge (p < 0.001), those without a previous DP/DMH experience during the COVID-19 pandemic (p < 0.001) or during the last triennium (2020-2023) (p < 0.001), those without DP/DMH knowledge (p < 0.001) or experience (p < 0.001) or without DP/DMH education/training (p < 0.001) and those working in the Southern regions (p = 0.007).

However, when asked about their routinary use of a set of technological tools in their not-professional life, most mental health professionals declared a frequent and regular use of computers (78.6%), 88% e-mails, 95.1% Internet, 95% smartphones, 83.7% apps). Only the tablet was the lesser frequently reported (only in 32.6% of the total sample). While, when asked about their routinary use of the same technological tools in their professional life, most mental health professionals declared much more frequently to use computers (90.2%), Internet (87.5%), and e-mails (86.7%). Furthermore, among the most frequently reported preferred technological device for delivering DMH/DP interventions, most mental health professionals more frequently use a telephone (46.1%), followed by e-mails (27.4%), video-conference tools such as Skype, Facetime, Zoom (24%), chats such as WhatsApp, Telegram, Messenger (22.8%), smartphones and/or tablets (18%), dedicated online platforms (12.1%), Social Networks such as Facebook, Twitter, etc. (3.9%), online forum (3.1%), and virtual rooms such as SecondLife, metaverse (2.1%) (**Table 3**).

**Table 3:** Second section - clinical experience across all professional categories.

How much more is probable that you recommend one of the following therapeutic interventions to your patient?	Total sample (N = 1,389), Me	M.D. Psychiatrists (N = 340), Me	M.D. Psychiatry trainees (N = 377), Me	Psychologists (N = 328), Me	Mental health professionals working in psychiatric education and/or rehabilitation (N = 344), Me
Face-to-face (in-person)	5.0	5.0	5.0	5.0	5.0
Web-based therapist-assisted synchronous interventions (e.g., e-mail, instant messaging, videoconferencing)	3.0	3.0	3.0	3.0	3.0
Web-based asynchronous interventions (e.g., self-help, computer-assisted, software-based)	2.0	2.0	2.0	2.0	2.0
Smartphone-based interventions (e.g., apps)	2.0	2.0	2.0	2.0	2.0
How much do you like the idea to provide DP/DMH interventions in your workplace?	3.0	3.0	3.0	3.0	3.0
How frequently do you use the following digital tools in your personal life?					
Computer	4.0	5.0	5.0	5.0	4.0
E-mail	5.0	5.0	5.0	5.0	5.0
Internet	5.0	5.0	5.0	5.0	5.0
Smartphone	5.0	5.0	5.0	5.0	5.0
Apps	5.0	5.0	5.0	5.0	5.0
Tablet	3.0	3.0	2.0	2.0	3.0
How frequently do you use the following digital tools in your professional life?					
Computer	5.0	5.0	5.0	5.0	5.0
E-mail	5.0	5.0	5.0	5.0	5.0
Internet	5.0	5.0	5.0	5.0	4.0
Smartphone	4.0	4.0	4.0	4.0	4.0
Apps	3.0	3.0	3.0	3.0	3.0
Tablet	1.0	1.0	1.0	1.5	2.0
How frequently do you use the following digital tools to deliver DP/DMH interventions to your patients?					
E-mail	2.0	3.0	2.0	2.0	2.0
Audio-conference	2.0	2.0	1.0	2.0	1.0
Video-conference	2.0	3.0	2.0	3.0	2.0
Online platforms	1.0	2.0	1.0	2.0	1.0
Online forum	1.0	1.0	1.0	1.0	1.0
Chats	2.0	2.0	1.0	3.0	2.0
Social Networks	1.0	1.0	1.0	1.0	1.0
Smartphones and/or Tablets	2.0	2.0	1.0	2.0	2.0
Virtual Rooms (e.g., Second Life)	1.0	1.0	1.0	1.0	1.0
Phone	3.0	4.0	3.0	3.0	3.0

Me, median score based on a 5-point Likert scale.

### 3.3 The digitalization score (DIGi index)

The average mean DIGi index was 9.9 (± SD = 3.5), without any sex-based differences (p = 0.112). Significant higher DIGi scores were found among mental health professionals who declared a pre-COVID-19 practical experience (p < 0.001) and clinical practice during the last 3 years in delivering DMH/DP (p < 0.001), by suggesting a COVID-19-related effect in boosting the digitalization process. Higher DIGi scores were found among psychiatrists and psychologists compared to psychiatry trainees (both with p<0.001) and PRT/PE (both with p<0.001) [F(3,1385)=78.403, p<0.001], by suggesting differences across mental health professionals to be further explored. While there are no statistically significant DIGi differences between psychiatrists and psychologists (p = 0.185). Coherently with one of the research hypotheses, significant higher DIGi scores were found among mental health professionals working in private outpatient services compared to all other job settings (all with p = 0.001). However, contrary to our hypothesis, those mental health professionals working in not-university public hospitals displayed higher DIGi scores compared to university public hospitals (p = 0.002) and private clinics (p < 0.001) [F(5,1383) = 20.362; p < 0.001]. Significant higher DIGi scores were found among professionals who regularly practice psychotherapy compared to other professionals [F(2,1386)=96.126; p < 0.001]. While no statistically significant differences were found depending on the type of psychotherapy training (p = 0.410). Significant higher DIGi scores were found among those mental health professionals working with colleagues with DP/DMH clinical practice [F(2,1386)=62.908; p < 0.001] and colleagues with enough knowledge in DP/DMH [F(2,1386)=20.500; p < 0.001], by suggesting that this external/environmental factor could be targeted in the digitalization implementation. Consequently, significant higher DIGi scores were found among those mental health professionals who much more likely recommend DP/DMH interventions to their patients [F(2,1386) = 55.161; p < 0.001], those more knowledgeable on DP/DMH [F(2,1386) = 20.317; p < 0.001], with a clinical experience on DP/DMH interventions [F(2,1386) = 44.877; p < 0.001], with a good level of DP/DMH education and training [F(2,1386) = 8.782; p < 0.001], those who manifested an interest in providing DP/DMH interventions in case of job opportunity [F(2,1386) = 14.470; p < 0.001]. While no significant DIGi differences were found among those who preferred face-to-face versus digital modality [F(2,1386) = 0.877; p = 0.067](**Table 4**). Finally, significant lower DIGi scores were found among mental health professionals coming from central regions compared to those working in the Italian northern regions, by suggesting a differential trend in digitalization across Italian regions, coherently with our initial hypothesis (p = 0.004).

**Table 4:** Socio-demographic characteristics of the sample, across all three DIGi groups.

	DIGi index M (SD)	Low DIGi (N = 661) N (%)	Moderate DIGi (N = 629) N (%)	High DIGi (N = 99) N (%)	P-values
Sex					
Males	10.1 (3.5)	442 (66.9%)	479 (76.2%)	66 (66.7%)	χ2 = 14.508; **p < 0.001**
Females	9.7 (3.6)	219 (33.1%)	150 (23.8%)	33 (33.3%)	
Professional category					
MD Psychiatrists	11.1 (3.2)	110 (16.6%)	201 (32.0%)	29 (29.3%)	χ2 = 183.598; **p < 0.001**
MD Psychiatry Trainees	8.6 (3.0)	252 (38.1%)	116 (18.4%)	9 (9.1%)	
Psychologists	11.6 (3.5)	90 (13.6%)	189 (30.0%)	49 (49.5%)	
PTR/PE	8.8 (3.3)	209 (31.6%)	123 (19.6%)	12 (12.1%)	
Job setting					
Public setting	9.7 (3.3)	515 (77.9%)	466 (74.1%)	51 (51.5%)	χ2 = 31.446; **p < 0.001**
Private setting	10.6 (3.9)	146 (22.1%)	163 (25.9%)	48 (48.5%)	
Geographical Zone					
Northern Italy	10.3 (3.4)	162 (24.5%)	184 (29.3%)	28 (28.3%)	χ2 = 5.567; p = 0.234
Central Italy	9.6 (3.5)	251 (38.0%)	205 (32.6%)	33 (33.3%)	
Southern Italy	10.0 (3.6)	248 (37.5%)	240 (38.2%)	38 (38.4%)	
Psychotherapy training					
none	9.1 (3.2)	509 (77.0%)	324 (51.5%)	31 (31.3%)	χ2 = 154.381; **p < 0.001**
yes, no current practice	9.8 (3.6)	41 (6.2%)	36 (5.7%)	4 (4.0%)	
yes, current practice	11.7 (3.3)	111 (16.8%)	269 (42.8%)	64 (64.6%)	
pre-COVID19 DP/DMH experience					
yes	12.8 (2.9)	28 (4.2%)	112 (17.8%)	40 (40.4%)	χ2 = 123.801; **p < 0.001**
no	9.5 (3.4)	633 (95.8%)	517 (82.2%)	59 (59.6%)	
Last 3 years DP/DMH experience					
yes	13.9 (1.9)	6 (0.9%)	297 (47.2%)	93 (93.9%)	χ2 = 563.038; **p < 0.001**
no	8.4 (2.7)	655 (99.1%)	332 (52.8%)	6 (6.1%)	
DP/DMH suggestion to patients					
no	9.1 (3.0)	656 (99.2%)	507 (80.6%)	4 (4.0%)	χ2 = 591.148; **p < 0.001**
yes	14.6 (2.0)	5 (0.8%)	122 (19.4%)	95 (96.0%)	
DP/DMH knowledge					
no	9.1 (3.1)	641 (97.0%)	452 (71.9%)	28 (28.3%)	χ2 = 318.673; **p < 0.001**
yes	13.6 (2.6)	20 3.0%)	177 (28.1%)	71 (71.7%)	
DP/DMH clinical practice among colleagues					
no	9.1 (3.0)	660 (99.8%)	502 (79.8%)	11 (11.1%)	χ2 = 535.134; **p < 0.001**
yes	14.7 (2.0)	1 (0.2%)	127 (20.2%)	88 (88.9%)	
DP/DMH knowledge among colleagues					
no	8.8 (2.9)	645 (97.6%)	424 (67.4%)	4 (4.0%)	χ2 = 492.025; **p < 0.001**
yes	13.8 (2.4)	16 2.4%)	205 (32.6%)	95 (96%)	
DP/DMH clinical experience					
no	9.2 (3.1)	653 (98.8%)	478 (76%)	30 (30.3%)	χ2 = 342.645; **p < 0.001**
yes	14.0 (2.3)	8 (1.2%)	151 (24.0%)	69 (69.7%)	
DP/DMH enough training					
no	9.7 (3.4)	652 (98.6%)	577 (91.7%)	70 (70.7%)	χ2 = 116.918; **p < 0.001**
yes	13.6 (2.9)	9 (1.4%)	52 (8.3%)	29 (29.3%)	
Face-to-face interventions					
no	9.3 (3.6)	56 (8.5%)	38 (6.0%)	7 (7.1%)	χ2 = 2.830; p = 0.243
yes	10.0 (3.5)	605 (91.5%)	591 (94.0%)	92 (92.9%)	
Interest in providing DP/DMH in case of job opportunity					
no	9.2 (3.2)	484 (73.2%)	351 (55.8%)	24 (24.2%)	χ2 = 105.311; **p < 0.001**
yes	11.2 (3.6)	177 (26.8%)	278 (44.2%)	75 (75.8%)	

All significant p-values are provided in bold.

When we classified mental health professionals depending on the three DIGi categories, most of the sample reported a low (47.6%; N = 661) or moderate DIGi index (45.3%; N = 629). Only 7.1% of them displayed a high DIGi index (N = 99) (**Table 4**). Three DIGi categories are homogeneously distributed across three Italian geographical areas (p = 0.234). Coherently with our hypothesis, a low DIGi was much more likely observed among female mental health professionals compared to the male counterpart (p < 0.001), among younger mental health professionals (p < 0.001) and those with a lower number of years of clinical experience (p < 0.001). A moderate DIGi index was significantly displayed by mental health professionals who currently practice psychotherapy (both psychologists and physicians) in their clinical practice (p < 0.001) and psychologists (p < 0.001), by suggesting that psychologists and psychotherapists probably should not be prioritized in the digitalization implementation at the preliminary stage. A high DIGi score was significantly found among mental health professionals working in private settings (p < 0.001), those who regularly provided DP/DMH interventions before the COVID-19 pandemic (p < 0.001) and during the last triennium (2020-2023) (p < 0.001), by supporting the hypothesis that COVID-19 factor increased digitalization process but also working in private settings. Obviously, those mental health professionals belonging to the high DIGi category are those significantly more prone to propose DP/DMH interventions to their patients (p < 0.001), more knowledgeable on DP/DMH interventions (p < 0.001) and more trained on DP/DMH interventions (p < 0.001), by supporting that education/training factor influences the level of digitalization. Interestingly, those mental health professionals belonging to low DIGi category are those more interested in providing DP/DMH interventions in case their jobs allowed them to provide a digital intervention (p < 0.001)(**Table 4**).

According to the multivariate linear regression model, DIGI score was positively predicted by a pre-COVID-19 clinical experience in DMH/DP (B=0.764; 95%CI=0.440-1.088; p<0.001], a post-COVID-19 clinical experience in DMH/DP (B=3.372; 95%CI=3.087-3.657; p<0.001], having colleagues who know DMH/DP (B=2.441; 95%CI=2.135-2.747; p<0.001], having colleagues who deliver DMH/DP in their clinical practice (B=1.781; 95%CI=1.422-2.139; p<0.001], having experience in deliver DMH/DP (B=0.629; 95%CI=0.238-1.019; p = 0.002], knowing DMH/DP interventions (B=0.629; 95%CI= 0.272-0.986; p < 0.001] and higher age (B=0.036; 95%CI=0.025- 0.046; p<0.001]. While working in a public job setting, predicted negatively the DIGi index (B=-0.325; 95%CI=(-0.563)-(-0.087); p = 0.007]. These variables statistically significantly predicted DIGi score (F(8, 1380)=401.543, p<0.001, R2 = 0.699) (**Table 5**).

**Table 5:** Multiple linear regression with DIGI index (as dependent variable).

	B	SE	β	t	P-value	95%IC lower limit	95%IC upper limit	Tolerance	VIF
(*constant*)	6.928	0.250		27.755	**<0.001**	6.439	7.418		
DP/DMH last 3 years experience, yes	3.372	0.145	0.433	23.227	**<0.001**	3.087	3.657	0.626	1.598
DP/DMH pre-COVID-19 experience, yes	0.764	0.165	0.073	4.622	**<0.001**	0.440	1.088	0.872	1.147
DP/DMH knowledge by colleagues, yes	2.441	0.156	0.291	15.657	**<0.001**	2.135	2.747	0.629	1.589
DP/DMH practice by colleagues, yes	1.781	0.183	0.184	9.746	**<0.001**	1.422	2.139	0.613	1.631
DP/DMH clinical practice, yes	0.629	0.199	0.066	3.158	**0.002**	0.238	1.019	0.494	2.024
DP/DMH knowledge, yes	0.629	0.182	0.071	3.454	**<0.001**	0.272	0.986	0.521	1.920
age (in years)	0.036	0.005	0.102	6.694	**<0.001**	0.025	0.046	0.943	1.061
public job setting	-0.325	0.121	-0.040	-2.678	**0.007**	-0.563	-0.087	0.956	1.046

SE, Standard Error; DP, Digital Psychiatry; DMH, Digital Mental Health. In bold significant p-values.

## 4 Discussion

**Figure 1:**
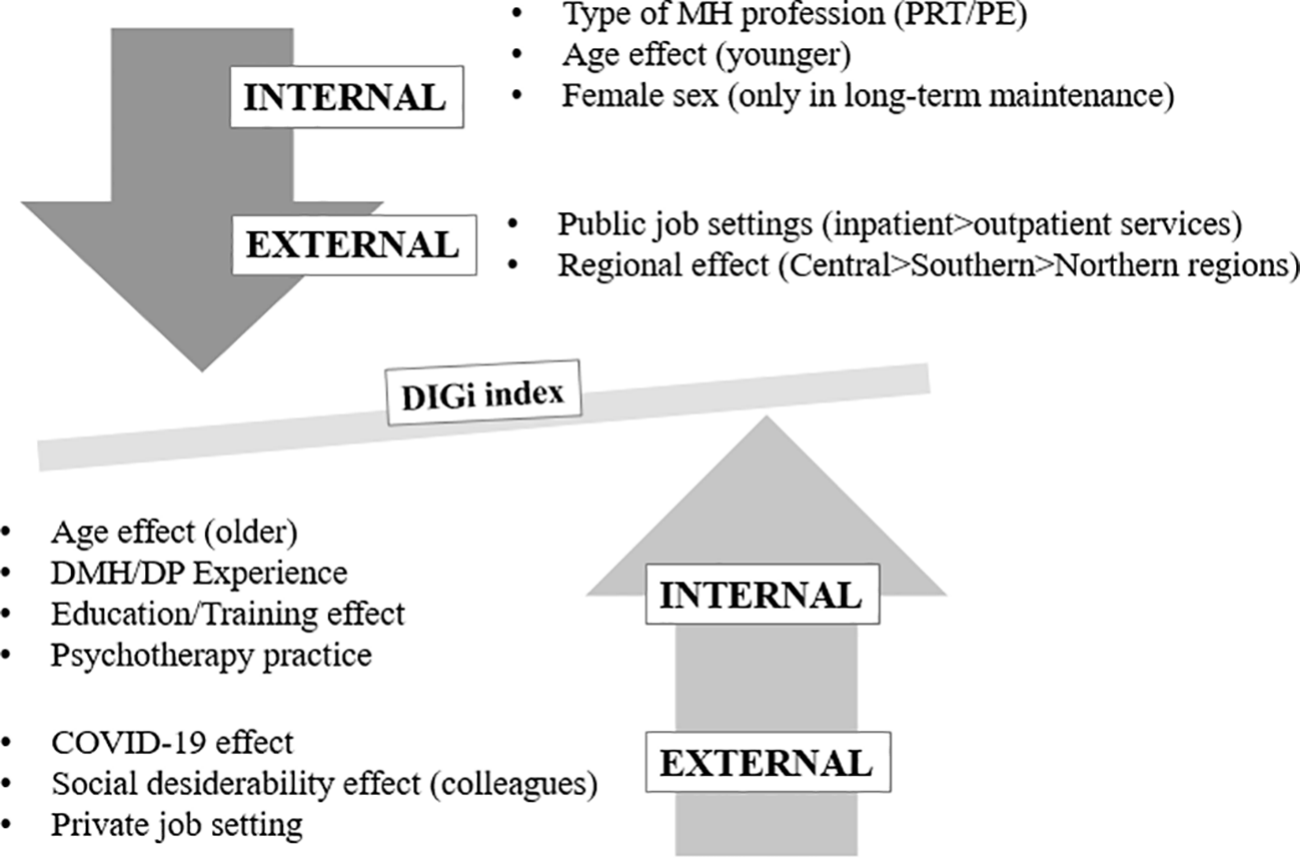
Internal/external factors influencing the DIGi index. MH, Mental Health; DMH, Digital Mental Health; DP, Digital Psychiatry; PRT, psychiatric rehabilitation technicians; PE, professional educators.

To the best of our knowledge, the current multicentric Italian-based study provides a first overview on the current digitalization level of mental health professionals and services in the Italian context. The study collected a good representative sample of mental health professionals, recruited from both public and private settings, and coming from all three geographical Italian areas. Our sample was constituted by relatively young participants comprising all mental health professional categories (physicians, psychologists and PRT/PE), recruited at different levels of psychiatry and/or psychotherapy training and owning different levels of clinical expertise. The study investigated the overall level of pre- and post-COVID-19 clinical experience in delivering DP/DMH interventions, the level of knowledge, expertise and practice in routine clinical practice in DP/DMH, as well as the general predisposition/openness to the digitalization of mental health and care among Italian mental health professionals, by identifying if any internal and/or external factors could influence these variables and the overall digitalization level (**Figure 1**). Our findings could inform training institutions and policy-makers about next steps to be prioritized in implementation strategies of digitalization of mental health systems and professionals, by clearly identifying which needs should be addressed considering both internal and external variables.

Overall, most of the sample declared the lack of a pre- (before 2020) and post-COVID-19 (triennium 2020-2023) clinical experience in DMH/DP. In both conditions, critical internal factors are represented by the youngest age and the poorest clinical experience in mental health and care in general (i.e. not specific for DP/DMH). Furthermore, critical external factors are represented by the public job setting and the type of professional category, being PRT, PE and psychiatry trainees, those professionals who declared the poorest clinical experience in DMH/DP interventions at all, independently by the COVID-19 outbreak. In our sample, female professionals are those who much more likely displayed the lowest level of post-COVID-19 clinical experience in DP/DMH interventions, by suggesting sex-based differences in the proneness in maintaining the delivery of DMH/DP over the time. One could argue that while there are no significant sex-based differences in providing DMH/DP interventions in case of critical contingent situations (i.e., COVID-19 outbreak), the maintenance of the digitalization proneness over the time could be much more likely influenced by the sex. Therefore, our findings should specifically guide policy makers and national initiatives to preferentially target female professionals in order to strengthen and consolidate good clinical practice in DMH, particularly in those mental health services which were already digitally implemented during the COVID-19 outbreak. Furthermore, our results reported that mental health professionals working in the southern Italian regions reported the poorest clinical experience in DMH/DP during the post-COVID19 triennium 2020-2023. This data could be supposed to be explained by the lack of adequate infrastructures, other limiting financial resources or by other knowledge/cultural-based geographical factors. Although these findings should be more deeply investigated to draw definitive conclusions, one could suppose that DMH/DP implementation in Italy could be geographically sensitive. In addition, our findings also documented another significant critical determinant represented by the lack of formal training and the overly poor knowledge in DMH/DP which influence the poorest clinical practice in DMH/DP by most participating mental health professionals. Indeed, these findings are coherent with previous published literature. The COVID-19 pandemic revealed a dramatic poor/absent digitalization within all mental health services worldwide, by pointing out the need to implement digitalization process by involving all levels of mental health infrastructures and professionals (1, 2, 24). Indeed, many mental health services and professionals were forced to implement TP, in routine clinical practice, as a needed alternative to the traditional in-person approach, only due to the COVID-19 outbreak. This forced digitalization process was not accompanied by a strong motivational process as well as by adequate training and education in DMH/DP among mental health professionals. Consequently, most mental health professionals declared to have forcibly accepted and temporarily implemented the digital modality which was mainly provided as a provisional or complimentary tool, by rapidly returning back to traditional face-to-face therapeutic modalities when contingencies were overcome (24, 25).

Moreover, according to our study, only one fifth of the sample reported to own enough knowledge in DMH/DP, while only few mental health professionals displayed a sufficient level of clinical experience/expertise in DMH/DP. Therefore, it seems that the Italian mental health professionals are overly poor both in DMH knowledge and clinical experience, independently by the COVID-19 factor, the geographical area, socio-demographic variables as well as the type of professional category. However, this picture appeared to be influenced by a set of potential influencing factors, as detailed below. The highest level of DMH/DP knowledge and clinical experience was documented in those mental health professionals working in private settings. Our main hypothesis about this difference could be more likely explained by differential financial resources, infrastructures, availability of technological tools, as well as a lower financial interest in the capillary implementation of DMH/DP within public mental health services due to the highest number of patient population (compared to the public settings). Therefore, policy makers should take into account the need to implement financial resources in terms of both equipment and funding education/training resources at public level, in order to facilitate the digitalization beyond private settings and psychotherapy. Another determinant is represented by the working context and the level of knowledge and experience in DMH/DP by other colleagues, by supporting the hypothesis that a stimulating and innovative work environment could positively influence (and motivate) the digitalization of mental health care and practice. Consequently, at institutional level, it could be beneficial to favor knowledge and education/training in academic settings, as well as during the clinical training practice. Further significant determinants are represented by the age factor and the number of years of clinical experience in mental health care. Our findings found the lowest level of knowledge and expertise in DMH/DP among the youngest professionals who indeed mostly belong to the digital natives’ generation. This finding could be explained by the lack of formal training since academic studies (reported in only 6.5% of the sample). In fact, most of the youngest professionals are declared to have acquired some knowledge mainly through a self-learning modality without mentorship or supervision by senior professionals. Coherently, most of the youngest professionals preferred to recommend face-to-face interventions rather than a digital approach in their clinical practice. Our findings documented that younger and less experienced mental health professionals are those less skilled, less trained and who less likely provide DMH/DP interventions in their clinical practice. Again, these findings support the need to prioritize the education/training needs and gaps in DMH/DP in academic settings to favor the digitalization proneness of younger professionals. Some authors suggested that poor clinicians’ motivation and interest in implementing and maintaining DMH/DP in their clinical practice, could be due to a poor theoretical and practical training/experience in the field of DMH/DP but also an age- or generation-related factor (e.g., belonging to the digital natives’ vs. the digital immigrants’ generation) (26). Coherently, the new/younger generation of clinicians (i.e., students, psychiatry trainees and young mental health professionals), owning an overall greater technology/digital literacy and readiness, should be more prone to provide DMH/DP interventions to their patients, compared to the senior mental health professionals (27). However, some studies indeed reported that younger mental health professionals, less likely have the opportunity to receive a formal or informal clinical/practical experience, an official training curriculum in DP/DMH, as well as a formal guided supervision by their (senior) mentors (generally lesser skilled in DMH/DP interventions) (28–31). Hence, this situation could potentially reduce the chance to promote DMH/DP to their patients, to incentivize younger professionals in being engaged in DMH/DP interventions, independently by their belonging to the most digital generation (28, 30, 31).

Therefore, in our study, although no specific internal/external factors seemed to influence the highest/lowest chance to receive a formal training on DMH/DP, one could argue that both the lack of DMH/DP knowledge (indeed depending on the level of training/education received) and the lack/poor education and training (depending on the lack of a formal university and post-doc training program in DMH/DP) might influence the lowest proneness to DMH by the youngest generation. The knowledge level (influenced by education and training) could also influence the level of clinicians’ awareness and perception about the effectiveness of DMH/DP interventions and, indirectly, influence the clinicians’ proneness in providing (and recommending) digital interventions rather than in-person modalities. In fact, one of our hypotheses could support the idea that it is not only needed to guarantee a basic level of education and training in DMH/DP but also provide an advanced and specialized training about all DMH/DP differential interventions and evidence-based practices in mental health care. In this regard, our findings clearly demonstrated that also among those mental health professionals who are more prone to recommend DMH/DP interventions, there is a limited preference (i.e., the synchronous modality with the support of a therapist represents the most preferred modality offered and recommended by Italian clinicians). Indeed, on one hand, this finding could suggest that Italian mental health professionals are overly more prone to integrate digital solutions only within the context of a concomitant traditional (in-person) practice. On the other hand, one could argue that this preference is mainly mediated by the lack of advanced training in DMH/DP. In order to corroborate these hypotheses, further longitudinal studies should be conducted by evaluating the effectiveness of a (basic versus advanced) training among all mental health professionals over the time. Overall, despite the abovementioned limiting factors in the digitalization process of mental health and care, most of the mental health professionals displaying the lowest DMH/DP experience, knowledge, training/education and those working in job settings with inexperienced and not knowledgeable colleagues, are those who declared to be more prone to learn more about DMH/DP. Moreover, in our study, most mental health professionals were declared to be prone to digitally integrate their clinical practice in case formal (theoretical and practical) training is provided to them. This reassuring finding supports again the need to capillary and early implement a training and educational course since the university courses, as this could represent a significant driving factor in increasing an effective chance towards the digitalization process of mental health and care. These findings are indeed in line with previous literature. Education and training represent the core components of a digitalization process able to consolidate the process already started and sometimes accelerated by the COVID-19 outbreak (32–34). Training and education should be addressed to both mental health professionals and infrastructures/services. Education and training in DMH/DP should allow clinicians to acquire basic and advanced digital/technological notions and apply them in mental health practice (29). Furthermore, while more initiatives have been stimulated in the field of TP, a less prompt and effective intervention has been developed in other DMH/DP topics, such as asynchronous DP platforms, integrated DP platforms and clinics, m-mental Health, virtual reality-based (VR) and/or augmented Reality (AR)-based DP interventions, and so forth (13, 14, 27, 35). Therefore, a training program should include all digital mental health interventions and not only TP/telemedicine applications (13, 14). Education/training needs and gaps should be prioritized at the early stages of the process of digitalization implementation, by supporting strategies at institutional and academic level.

In our study, we also explored potential differences in the digitalization level (knowledge, experience, expertise) across all mental health professionals, by comparing their use of digital tools in both personal and professional life, to identify if any internal (not job-related) factors could influence the digitalization process in mental health care. Our findings clearly documented that most mental health professionals much more likely use digital devices and tools in their personal life than in their professional settings. Most mental health professionals appeared to own good digital and technological skills and competences. These findings could be explained by analyzing both external (institutional) and internal (individual) variables. External factors might comprise the type of job setting, the level of financial resources and technology equipment provided by the institution as well as the level of education and training supported (and financed) by the institution itself as well as all incentivizing and supporting initiatives to increase DMH/DP practice. Internal factors might comprise age- and sex-based factors, as already discussed above, or the level of knowledge and expertise in applying digital tools and interventions in their clinical practice, which could be mostly justified by the lack/poor personal education and training received by academia.

Overall, our study reported an Italian situation which is poorly digitalized in mental health and care, with mostly participating mental health professionals displaying a low DIGi index. In our Italian study, a low DIGi index is influenced by internal (e.g., female sex, youngest age, the lowest clinical experience, being a PRT/PE or a psychiatry trainee) and external determinants (e.g., public settings, university hospitals, the lowest level of education and training in DMH/DP). A moderate DIGi index is mainly found in both psychologists and psychiatrists, particularly those who regularly practice psychotherapy and work in private settings. Therefore, it seems that DMH in Italy would be preferentially practiced for offering psychotherapeutic interventions. The most common Italian settings in which are routinely provided DMH/DP interventions are private settings and not-university public settings, as well as those mental health services located in the Northern regions. Not a preferential type of psychotherapy seemed to emerge. The highest DiGi index was significantly predicted by social working influence (i.e., working with colleagues owning DP/DMH clinical practice and knowledge), previous consolidated knowledge, experience and expertise as well as education and training in DMH/DP.

Despite these interesting findings, our study displays a set of limitations which should be adequately discussed and integrated in the interpretation and generalization of our main results. Firstly, our sample is mainly represented by relatively young mental health professionals (lower than 50-years-old) which could indeed represent the current ‘real-world’ representativeness of Italian mental health professionals, given the recent massive retirement of many health professionals. Indeed, surprisingly, despite our preliminary hypothesis supporting the idea of an age- and generation-sensitive factor in favoring digital implementation of mental health care, our findings indeed found a worrying gap in DMH/DP clinical practice mainly by the youngest professionals, even though mainly belonging to the digital native’s generation. Conversely, according to our findings, senior mental health professionals were much more likely knowledgeable and experienced in providing DMH/DP interventions, and then they usually much more likely offer digital interventions in their clinical practice. Secondly, our sample mainly recruited male mental health professionals, with an unbalanced rate particularly among psychologists and PRT/PE, which could influence the generalizability of the sex-sensitive findings. This finding could suggest the need to increase the sample in order to investigate whether sex-based differences could represent a selection bias. Therefore, another limitation could be represented by potential selection bias due to voluntary survey participation. Thirdly, the cross-sectional design of the study does not allow to draw up definitive conclusions regarding the longitudinal trajectory or causal inference between the DIGi index and the predictive factors such as the impact of an educational and training program over the time, the change of job setting (from public to private and viceversa), the change in knowledge and expertise level, the age- and clinical experience in mental health care over the time, and so forth. Fourthly, our study did not investigate the characteristics of mental health systems of each mental health professionals, in terms of starting time of digital implementation, which types of digital interventions have been integrated and delivered, how many mental health professionals and their professional roles are present in each mental health system, which type of technological equipment were integrated, if any training and/or course was provided to mental health professionals before implementing digital solutions in clinical practice, and so forth. Moreover, our study did not specifically collect characteristics of public versus private settings across all Italian regions, by specifically identifying if there are geographical differences, also depending on the cultural proponeness by patients. In fact, we did not collect specifically characteristics of private versus public settings in terms of financial resources, sustainability and the level of technology equipment, in order to better understand which structural factors could determine the higher or lower digitalization level. Finally, the survey is constituted by self-reported tools which could potential infer the generalizability of results. However, despite the abovementioned limitations, our study provided the first picture of the Italian situation in terms of DMH by recruiting a representative sample of different mental health professionals, by including also those categories who provide psychiatric rehabilitative interventions which recently represent one of the most promising fields of the application of digital solutions (14), but also recruiting a good geographical representativeness of mental health professionals.

Our findings indicate further research directions to be deeply investigated but also provide a preliminary national direction by selecting a set of internal and external modifiable factors which could positively/negatively influence the digitalization process of mental health care in the Italian context. Further *post-hoc* analyses will be carried out to determine whether digital literacy, readiness, the level of acceptability and perceived feasibility by mental health professionals and their patients could also influence the Italian DIGi index in mental health care. National initiatives should firstly address education and training needs by the youngest mental health professionals, particularly those without a mentor/supervisor experienced in providing DMH/DP and in educating younger professionals in the digital clinical practice. Female professionals should be specifically targeted and investigated in consolidating initiatives able to strengthen that digitalization process already effective during the COVID-19 pandemic, as there is a sex-sensitive decreasing trend which should be promptly targeted, considering the further increasing number of health professionals in the Italian context. Another determinant to be addressed is represented by the structural and infrastructural opportunities offered by the job setting. Public settings at all levels (not-university and university) should pay attention to implement motivating and incentivizing digitalization programs, by offering available, sustainable and free training and educational courses to all their mental health professionals as well as build an open and not-judgmental environment facilitating the delivery of digital solutions and interventions. Other steps to be prioritized consisted in asking to policy makers national financial resources able to cover the gaps in equipment/technical tools at private settings, to incentivize resources in education/training since academic settings and in increasing knowledge among mental health professionals about DP/DMH beyond TP.

Future research studies could be designed in order to evaluate whether interventional studies specifically improving the level of knowledge, education and training of DMH/DP across all mental health professionals, by recruiting students and professionals since their academic studies could effectively improve the digital proneness towards the use of DMH/DP tools and interventions in clinical practice, as well as effectively favoring the increase of DIGi score across all mental health services in Italy. Other open questions which should be furtherly investigated should also include an analysis of effective costs/financial resources needed to maintain the continuity of DMH/DP in routine clinical practice, by evaluating whether there is an effect induced only by financial investment or rather other internal determinants (depending by the professional) which induce the choice of private settings in practicing DMH/DP rather than public contexts. Therefore, further longitudinal and interventional studies with periodic follow-up should be designed to detect which interventions at national level could be more effective in the short- and long-term and whether the proposed tailored interventions (considering internal/external factors here identified) could represent an effective strategy to consolidate the digitalization of mental health care.

## Data Availability

The raw data supporting the conclusions of this article will be made available by the authors, without undue reservation.
