# Supplementary material for: Digital mental health in Italy: findings from the multicentric DIGIT-PSY study
Source: Front Psychiatry. 2025 Aug 29;16:1642455. doi: 10.3389/fpsyt.2025.1642455 (PMC12426103; doi:10.3389/fpsyt.2025.1642455)
Supplement: Supplementary file 1 [file Supplementaryfile1.pdf]

Questionario per i professionisti della Salute Mentale

Sezione socio-demografica

Le chiediamo di compilare le seguenti informazioni anagrafiche:

Indichi il suo sesso: ◯ Maschio ◯ Femmina ◯ Altro (specificare: _________)

Indichi la sua età anagrafica (in anni): _____________

Indichi la sua professione, tra le di seguito elencate:

◯ Medico specialista in Psichiatria

◯ Medico specializzando/in formazione specialistica in Psichiatria

◯ Medico specialista in Psichiatria dottorando

◯ Medico specialista in Psichiatria assegnista di ricerca/ricercatore universitario

◯ Medico specialista in Psichiatria professore universitario

◯ Psicologo/a

◯ Psicologo/a psicoterapeuta

◯ Educatore Professionale

◯ Tecnico della Riabilitazione Psichiatrica

Indichi il suo principale setting di lavoro, tra le di seguito elencate:

◯ pubblico ospedaliero

◯ pubblico ospedaliero universitario

◯ pubblico territoriale

◯ privato libera professione ambulatoriale

◯ privato ospedaliero

◯ universitario

In merito alla specializzazione in psicoterapia, scelga una delle seguenti:

◯ Non ho mai frequentato una scuola di specializzazione in psicoterapia

◯ Ho frequentato una scuola di specializzazione in psicoterapia ma non la pratico nella mia attività clinica

◯ Ho frequentato una scuola di specializzazione in psicoterapia e la pratico nella mia attività clinica

In merito alla specializzazione in psicoterapia, scelga una dei seguenti orientamenti:

◯ Ho frequentato una scuola di specializzazione in psicoterapia ad orientamento cognitivo-comportamentale

◯ Ho frequentato una scuola di specializzazione in psicoterapia ad orientamento sistemico-relazionale e/o familiare

◯ Ho frequentato una scuola di specializzazione in psicoterapia ad orientamento psicodinamico e/o psicoanalitico

Indichi la regione in cui esercita la sua professione: _________________

Indichi la città in cui esercita la sua professione: ____________________

Indichi il numero di anni di esperienza clinica complessivi nell'ambito della Salute Mentale (compreso il periodo di formazione): ____

Sezione esperienza clinica

Le chiediamo di compilare la seguente sezione che riguarda sia la sua esperienza clinica in ambito di erogazione di interventi di psichiatria digitale:

1= mai; 2 = raramente; 3 = qualche volta; 4 = spesso; 5 = sempre					
Prima della Pandemia da COVID-19 ha mai erogato interventi di psichiatria digitale?	Si		No		
Negli ultimi 3 anni quanto ha erogato interventi di psichiatria digitale?	1	2	3	4	5
Se ha risposto alla precedente domanda mai (1), raramente (2) o qualche volta (3), quali sono le sue principali motivazioni? (si prega di scegliere una opzione)<br>◯ Non saprei come applicare gli interventi digitali alla mia pratica clinica<br>◯ Non ritengo di aver ricevuto una formazione sufficiente per applicare gli interventi digitali alla mia pratica clinica<br>◯ Considero gli interventi digitali non efficienti (es. non ritengo che consentano l'utilizzo adeguato delle risorse a disposizione per l'erogazione dell'intervento terapeutico)<br>◯ Considero gli interventi digitali non efficaci nella pratica clinica<br>◯ Trovo gli interventi digitali molto impersonali<br>◯ Non considero gli interventi digitali abbastanza sicuri<br>◯ Non considero gli interventi digitali eticamente accettabili<br>◯ Trovo gli interventi digitali molto costosi					
Quanto sono utilizzati gli interventi di psichiatria digitale dai suoi colleghi nel suo ambiente di lavoro?	1	2	3	4	5
Quanto è diffusa la conoscenza degli interventi di psichiatria digitale tra i suoi colleghi nel suo ambiente di lavoro?	1	2	3	4	5
Ha mai consigliato un intervento di psichiatria digitale ad un suo paziente?	1	2	3	4	5
1= quasi nulla; 2 = poco; 3 = nella media; 4 = discretamente; 5 = moltissimo					
Quanto conosce la psichiatria digitale?	1	2	3	4	5
Quanta esperienza clinica ha acquisito nell'ambito degli interventi di psichiatria digitale?	1	2	3	4	5
Quanto ritiene di aver ricevuto una formazione nell'ambito degli interventi di psichiatria digitale?	1	2	3	4	5
1= mai; 2 = raramente; 3 = qualche volta; 4 = spesso; 5 = sempre					
Quanto è probabile che lei possa raccomandare uno dei seguenti interventi terapeutici ad un suo paziente?					
Face-to-face (di persona)	1	2	3	4	5
Interventi web-based con associato il supporto del terapeuta<br>(es. email, instant messaging, videoconferencing)	1	2	3	4	5
Interventi web-based senza il supporto del terapeuta<br>(es. self-help, computerizzati, software-based)	1	2	3	4	5

Interventi smartphone-based (es. apps)	1	2	3	4	5
Quanto le piace l'idea di poter erogare interventi di Psichiatria Digitale nel suo ambiente di lavoro?	1	2	3	4	5
Quanto frequentemente utilizza uno dei seguenti strumenti tecnologici nella sua vita (al di fuori dell'ambito professionale)?					
Computer	1	2	3	4	5
E-mail					
Internet	1	2	3	4	5
Smartphone	1	2	3	4	5
Apps	1	2	3	4	5
Tablet	1	2	3	4	5
Quanto frequentemente utilizza uno dei seguenti strumenti tecnologici nel suo ambiente di lavoro per motivi lavorativi?					
Computer	1	2	3	4	5
E-mail	1	2	3	4	5
Internet	1	2	3	4	5
Smartphone	1	2	3	4	5
Apps	1	2	3	4	5
Tablet	1	2	3	4	5
Quanto frequentemente utilizza uno dei seguenti strumenti per erogare interventi di psichiatria digitale ai suoi pazienti?					
E-mail	1	2	3	4	5
Audio-conference (es. Skype, Facetime, Zoom)	1	2	3	4	5
Video-conference (es. Skype, Facetime, Zoom)	1	2	3	4	5
Piattaforme online	1	2	3	4	5
Forum online	1	2	3	4	5
Chat (es. Whatsapp, Telegram, Messenger, ecc.)	1	2	3	4	5
Social Networks (es. Facebook, Twitter, Instagram, LinkedIn, ecc.)	1	2	3	4	5
Smartphone e tablets	1	2	3	4	5
Virtual Rooms (es. Second Life)	1	2	3	4	5
Telefono	1	2	3	4	5

Sezione Digital Literacy

1= molto facile; 2 = facile; 3 = difficile; 4 = molto difficile

Quanto è facile o difficile per lei …

1.Utilizzare la tastiera di un computer (es. scrivere al computer)	1	2	3	4
2.Utilizzare il mouse (es. cliccare o spostare il cursore del mouse)	1	2	3	4
3.Usare i pulsanti o i links e hyperlinks sui siti web?	1	2	3	4

Quando cerchi su Internet informazioni, quanto è facile o difficile per lei …

4.Scegliere tra le informazioni che trovi	1	2	3	4
5.Utilizzare le parole o i criteri di ricerca per trovare le informazioni che sta cercando	1	2	3	4
6.Trovare le esatte informazioni che sta cercando	1	2	3	4
7.Decidere se le informazioni trovate siano attendibili o meno	1	2	3	4
8.Decidere se le informazioni che ha trovato sono scritte per interessi commerciali (es. siano scritte da persone che provano a vendere un prodotto)	1	2	3	4
9.Confrontare diversi siti web per vedere se forniscono le stesse informazioni	1	2	3	4
10.Decidere se le informazioni che trova vadano bene sulla base di quello che sta cercando	1	2	3	4
11.Applicare le informazioni che trova alla sua vita quotidiana	1	2	3	4
12.Usare le informazioni che trova per prendere decisioni sulla sua salute (es. sulla nutrizione, farmaci o decidere se chiedere un'opinione al tuo medico)	1	2	3	4

Quando cerca informazioni su Internet, quanto spesso le capita di…

13.Perdersi sui siti web o su Internet?	1	2	3	4
14.Non riuscire a ritornare nella pagina precedente di ricerca?	1	2	3	4
15. Cliccare su qualche contenuto che non risulta attinente alla tua ricerca?	1	2	3	4

Quando compone un messaggio (ad esempio sulle chat, sui social media, ecc.) quanto è semplice…

16.Formulare la domanda o il pensiero in modo semplice e comprensibile?	1	2	3	4
17.Esprimere la sua opinione, pensieri o sentimenti mentre scrive?	1	2	3	4

18. Scrivere un messaggio in modo che la persona a cui lo sta scrivendo comprenda in modo esatto quello che sta comunicando a lui/lei?	1	2	3	4

Quando posta un messaggio (ad esempio sui social media, sui forum, ecc.) quanto spesso…

19.Trova difficile giudicare chi lo leggerà?	1	2	3	4
20.Condivide (intenzionalmente o non intenzionalmente) le sue informazioni personali (ad esempio, il suo nome o il suo indirizzo)?	1	2	3	4
21. Condivide (intenzionalmente o non intenzionalmente) le proprie informazioni personali/private a qualcuno?	1	2	3	4

Sezione Acceptability e Social Influence

1= fortemente in disaccordo; 2 = in disaccordo; 3 = né d'accordo né in disaccordo; 4 = d'accordo; 5 = fortemente d'accordo

1.Generalmente, gli interventi di psichiatria digitale sono un valido complemento ai servizi medici	1	2	3	4	5
2.Per problemi psichiatrici o psicoterapeutici o per le malattie mentali, è molto utile fornire informazioni al paziente tramite l'impiego di Internet o la psichiatria digitale	1	2	3	4	5
3.È possibile erogare un trattamento efficace a pazienti affetti da malattie mentali tramite Internet o interventi di psichiatria digitale	1	2	3	4	5
4.Superare i tempi di attesa per un appuntamento psichiatrico/psicologico/psicoterapico utilizzando Internet o la psichiatria digitale potrebbe essere un'opzione	1	2	3	4	5
5.La presa in carico e la stabilizzazione dopo una terapia in presenza da parte di uno psichiatra, psicologo o uno psicoterapeuta attraverso contatti via Internet, e-mail, o telefono può essere fattibile	1	2	3	4	5
6.Raccomanderei assolutamente ai miei pazienti che necessitano di un trattamento psichiatrico, psicologico o psicoterapico una terapia online, se il quadro clinico non lo controindica. (versione medico)<br>6.Per i miei pazienti, offrirei supporto online e proporrei interventi di psichiatria digitale tramite Internet o telefono (versione psicoterapeuta/psicologo/TRP)	1	2	3	4	5
7.Oltre ad una terapia face-to-face, è un'opzione fattibile associare un intervento psicoeducativo, psicosociale o complementare tramite Internet o interventi di psichiatria digitale.	1	2	3	4	5
8.Una terapia online per i disturbi mentali può funzionare in modo efficace solo se associata ad un contatto dal vivo con il terapeuta tramite videocalling, email o chat.	1	2	3	4	5

1= molto negativamente; 2 = negativamente; 3 = indifferentemente; 4 = positivamente; 5 = molto positivamente

Come pensi possano giudicarti i tuoi colleghi se tu decidessi di integrare interventi di psichiatria digitale nella tua pratica clinica quotidiana?	1	2	3	4	5
Come pensi possano giudicarti i tuoi pazienti, familiari, conoscenti se tu decidessi di integrare interventi di psichiatria digitale nella tua pratica clinica quotidiana?	1	2	3	4	5

Sezione Digital Readiness (Technology Readiness Scale 2.0./TRI 2.0)

1= fortemente in disaccordo; 2 = in disaccordo; 3 = né d'accordo né in disaccordo; 4 = d'accordo; 5 = fortemente d'accordo

1.Le nuove tecnologie contribuiscono ad una migliore qualità della vita.	1	2	3	4	5
2.La tecnologia mi dà maggiore libertà di movimento.	1	2	3	4	5
3.La tecnologia dà alle persone un maggiore controllo sulla propria vita quotidiana.	1	2	3	4	5
4.La tecnologia mi rende più produttivo nella mia vita personale.	1	2	3	4	5
5.Le altre persone mi chiedono aiuto per i nuovi mezzi tecnologici.	1	2	3	4	5
6.In generale, sono tra i primi nella mia cerchia di amici ad acquisire i più nuovi mezzi tecnologici appena escono.	1	2	3	4	5
7.Di solito riesco a capire i prodotti e servizi tecnologici appena usciti senza aver bisogno di chiedere aiuto ad altri.	1	2	3	4	5
8.Sono al passo con gli ultimi sviluppi tecnologici nelle mie aree di interesse.	1	2	3	4	5
9R. Quando ricevo supporto tecnologico da un fornitore di un prodotto o servizio high-tech, mi sento qualche volta come se stessi approfittando di qualcuno che ne sa molto più di me.	1	2	3	4	5
10R.Qualche volte penso che i sistemi tecnologici non siano stati creati per essere utilizzati dalla gente comune.	1	2	3	4	5
11R.Le linee di supporto tecnico non sono utili perché non spiegano le cose in termini che io comprendo.	1	2	3	4	5
12R.Non esiste un manuale di prodotti o servizi high-tech scritto in un linguaggio semplice.	1	2	3	4	5
13R.Le persone dipendono troppo dalla tecnologia per fare le cose da soli.	1	2	3	4	5
14 R. Troppa tecnologia distrae le persone a tal punto da diventare dannosa.	1	2	3	4	5
15R.La tecnologia riduce la qualità delle relazioni limitando le interazioni personali.	1	2	3	4	5
16R.Non mi sento a mio agio a lavorare in un posto che può essere raggiunto solamente online.	1	2	3	4	5

Sezione Satisfaction and Feasibility

1= fortemente in disaccordo; 2 = in disaccordo; 3 = né d'accordo né in disaccordo; 4 = d'accordo; 5 = fortemente d'accordo

1.Sono stato soddisfatto rispetto al mio orientamento nell'erogare l'intervento di psichiatria digitale.	1	2	3	4	5
2.Sono stato soddisfatto rispetto la qualità del servizio di psichiatria digitale erogato.	1	2	3	4	5
3. La qualità audio era accettabile.	1	2	3	4	5
4. La tecnologia (per quanto riguarda il processo tecnico che mi ha permesso di erogare l'intervento e non tanto i problemi tecnici che eventualmente ho riscontrato nell'erogare il servizio) mi ha distratto durante l'erogazione dell'intervento terapeutico.	1	2	3	4	5
5. L'impossibilità nel visitare fisicamente il mio paziente ha sicuramente compromesso il processo diagnostico.	1	2	3	4	5
6. Riesco in modo accurato ad accedere ai sintomi osservabili.	1	2	3	4	5
7. Non sono stato capace di osservare i dettagli dell'espressione facciale e dei movimenti del corpo del mio paziente e questo ha sicuramente compromesso la mia capacità di entrare in sintonia con il mio paziente.	1	2	3	4	5
8. Il rapporto tra clinico-paziente è stato compromesso dall'uso dell'intervento di psichiatria digitale.	1	2	3	4	5
9. Avrei preferito vedere il mio paziente di persona.	1	2	3	4	5
10. Le difficoltà tecniche hanno fatto perdere troppo tempo al processo.	1	2	3	4	5
11. La mia comunicazione con il paziente e/o il mio mettermi in contatto con il suo referente è stato compromesso dall'uso dell'intervento di psichiatria digitale.	1	2	3	4	5
12. In generale, il sistema era accettabile e facile da utilizzare.	1	2	3	4	5
13. Usare interventi di psichiatria digitale richiede più tempo rispetto agli interventi face-to-face.	1	2	3	4	5
14. Qualora avessi riscontrato qualche problema durante il servizio di erogazione dell'intervento di psichiatria digitale, avrei avuto la possibilità di chiedere un aiuto a qualcuno.	1	2	3	4	5
15. L'intervento di psichiatria digitale erogato oggi potrebbe aver migliorato la prognosi del mio paziente.	1	2	3	4	5

16. Il mio paziente è sembrato soddisfatto dal mio intervento di psichiatria digitale.	1	2	3	4	5
17. Il mio paziente ha espresso la volontà di usare nuovamente interventi di psichiatria digitale.	1	2	3	4	5
18. In generale, sono stato soddisfatto dell'intervento di psichiatria digitale che ho erogato.	1	2	3	4	5
19. Utilizzerei ancora interventi di psichiatria digitale per vedere i miei pazienti.	1	2	3	4	5
20. Raccomanderei ai miei colleghi di utilizzare interventi di psichiatria digitale.	1	2	3	4	5
21. La psichiatria digitale migliora l'efficienza clinica.	1	2	3	4	5
22. L'intervento di psichiatria digitale erogato in data odierna potrebbe aver reso più facile al mio paziente l'accesso alle cure.	1	2	3	4	5
23. L'intervento di psichiatria digitale erogato in data odierna potrebbe aver permesso al mio paziente l'accesso ai servizi più rapidamente rispetto ad un intervento erogato di persona.	1	2	3	4	5
24. Se l'intervento di psichiatria digitale non fosse stato disponibile oggi, il mio paziente avrebbe dovuto mettersi in viaggio per ricevere il servizio.	1	2	3	4	5
25. L'intervento di psichiatria digitale erogato in data odierna ha fatto risparmiare tempo al mio paziente	1	2	3	4	5
