# Supplementary material for: Digital mental health in Italy: findings from the multicentric DIGIT-PSY study
Source: Front Psychiatry. 2025 Aug 29;16:1642455. doi: 10.3389/fpsyt.2025.1642455 (PMC12426103; doi:10.3389/fpsyt.2025.1642455)
Supplement: Supplementary file 2 [file Supplementaryfile2.pdf]

Questionnaire for mental health professionals

Socio-demographic section

Please fill in the following personal details

Please enter your sex: ◯ Male ◯ Female ◯ Other (please specify:)

Please enter your age (in years): ___________________

Please indicate your profession from the list below:

◯ Specialist in Psychiatry
◯ Psychiatry Resident/Trainee
◯ Specialist in Psychiatry, PhD student
◯ Specialist in Psychiatry, research fellow/university researcher
◯ Specialist in Psychiatry, Professors of psychiatry
◯ Psychologist
◯ Psychologist and psychotherapist
◯ Professional Educator
◯ Psychiatric rehabilitation technician

Please indicate your main work setting from the list below:

◯ public hospital
◯ public university hospital
◯ pubblic community-based service
◯ private outpatient
◯ private hospital
◯ university

Regarding your psychotherapy training, please choose one:

◯ I have never attended a school of psychotherapy specialization
◯ I have attended a psychotherapy specialization school but I do not practice psychotherapy in my clinical activity
◯ I have attended a psychotherapy specialization school and I do practice psychotherapy in my clinical activity

Regarding your psychotherapy orientation, please choose one:

◯ Cognitive-behavioral
◯ Systemic-relational and/or family-oriented
◯ Psychodynamic and/or psychoanalytic

Region where you currently practice: ______________________________

City where you currently practice: _________________________________

Total number of years of clinical experience in Mental Health (including training period): ___

Clinical Experience

Please complete the following section regarding your clinical experience in delivering digital psychiatry interventions:

1 = Never 2 = Rarely 3 = Sometimes 4 = Often 5 = Always					
Before the COVID-19 pandemic, had you ever delivered digital psychiatry interventions?	Yes		No		
In the past 3 years, how often have you delivered digital psychiatry interventions?	1	2	3	4	5
If you answered "never" (1), "rarely" (2), or "sometimes" (3) to the previous question, what are your main reasons? (Please choose one)<br>◯ I wouldn't know how to apply digital interventions to my clinical practice<br>◯ I believe I have not received adequate training to apply digital interventions<br>◯ I consider digital interventions inefficient (e.g., they do not allow for optimal use of available resources for therapeutic delivery)<br>◯ I consider digital interventions ineffective in clinical practice<br>◯ I find digital interventions very impersonal<br>◯ I do not consider digital interventions sufficiently safe<br>◯ I do not consider digital interventions ethically acceptable<br>◯ I find digital interventions too expensive					
To what extent are digital psychiatry interventions used by your colleagues in your workplace?	1	2	3	4	5
How widespread is the knowledge of digital psychiatry interventions among your colleagues?	1	2	3	4	5
Have you ever recommended a digital psychiatry intervention to a patient?	1	2	3	4	5
1 = Not at all 2 = A little 3 = Average 4 = Quite a lot 5 = Very much					
How much do you know about digital psychiatry?	1	2	3	4	5
How much clinical experience have you gained in digital psychiatry interventions?	1	2	3	4	5
How much training do you believe you have received in this field?	1	2	3	4	5
1= never; 2 = rarely; 3 = sometimes; 4 = often; 5 = always					
How likely are you to recommend the following types of therapeutic interventions to your patients?					
Face-to-face (in person)	1	2	3	4	5
Web-based with therapist support (e.g., email, instant messaging, videoconferencing)	1	2	3	4	5
Web-based without therapist support (e.g., self-help, computer-based, software)	1	2	3	4	5

Smartphone-based (e.g., apps)	1	2	3	4	5
How much do you like the idea of delivering digital psychiatry interventions in your workplace?	1	2	3	4	5
How frequently do you use the following technologies in your personal (non-professional) life?					
Computer	1	2	3	4	5
E-mail					
Internet	1	2	3	4	5
Smartphone	1	2	3	4	5
Apps	1	2	3	4	5
Tablet	1	2	3	4	5
How frequently do you use the following technologies in your professional environment?					
Computer	1	2	3	4	5
E-mail	1	2	3	4	5
Internet	1	2	3	4	5
Smartphone	1	2	3	4	5
Apps	1	2	3	4	5
Tablet	1	2	3	4	5
How frequently do you use the following technologies to deliver digital psychiatry interventions to your patients?					
E-mail	1	2	3	4	5
Audio-conferencing (es. Skype, Facetime, Zoom)	1	2	3	4	5
Video-conferencing (es. Skype, Facetime, Zoom)	1	2	3	4	5
Online platforms	1	2	3	4	5
Online forums	1	2	3	4	5
Chats (e.g., Whatsapp, Telegram, Messenger, etc.)	1	2	3	4	5
Social Networks (e.g. Facebook, Twitter, Instagram, LinkedIn, etc.)	1	2	3	4	5
Smartphone and tablets	1	2	3	4	5
Virtual Rooms (e.g. Second Life)	1	2	3	4	5
Phone	1	2	3	4	5

Digital Literacy

1 = Very easy   2 = Easy   3 = Difficult   4 = Very difficult

How easy or difficult is it for you to…

1. Use a computer keyboard (e.g., type on a computer)	1	2	3	4
2. Use a mouse (e.g., click or move the cursor)	1	2	3	4
3. Use buttons or links and hyperlinks on websites	1	2	3	4

When searching for information on the Internet, how easy or difficult is it for you to…

4. Select relevant information from what you find	1	2	3	4
5. Use keywords or search criteria to find the information you're looking for	1	2	3	4
6. Find the exact information you're looking for	1	2	3	4
7. Judge whether the information you find is trustworthy or not	1	2	3	4
8. Determine whether the information is written for commercial purposes (e.g., by someone trying to sell a product)	1	2	3	4
9. Compare different websites to see if they provide the same information	1	2	3	4
10. Decide whether the information found fits your needs	1	2	3	4
11. Apply the information you find to your everyday life	1	2	3	4
12. Use the information to make decisions about your health (e.g., nutrition, medications, or whether to consult a doctor)	1	2	3	4

When searching for information on the Internet, how often do you…

13. Get lost on websites or on the Internet	1	2	3	4
14. Fail to return to the previous search page	1	2	3	4
15. Click on content that turns out to be unrelated to your search	1	2	3	4

When composing a message (e.g., in chats, on social media, etc.), how easy is it for you to…

16. Formulate a question or thought clearly and understandably	1	2	3	4
17. Express your opinions, thoughts, or feelings in writing	1	2	3	4
18. Write a message so the recipient fully understands what you are trying to communicate	1	2	3	4

When posting a message (e.g., on social media, forums, etc.), how often do you…

19. Find it hard to judge who will read it	1	2	3	4
20. Share personal information (e.g., your name or address), intentionally or unintentionally	1	2	3	4
21. Share private/personal information with others, intentionally or unintentionally	1	2	3	4

Acceptability e Social Influence

1 = Strongly disagree 2 = Disagree 3 = Neutral 4 = Agree 5 = Strongly agree

1. In general, digital psychiatry interventions are a valid complement to medical services	1	2	3	4	5
2. For psychiatric, psychotherapeutic problems or mental illnesses, it is very useful to provide information to the patient through the use of the Internet or digital psychiatry	1	2	3	4	5
3. It is possible to deliver effective treatment for patients with mental illness via the Internet or digital psychiatry interventions	1	2	3	4	5
4.Reducing waiting times for psychiatric/psychological/psychotherapy appointments through digital psychiatry or the Internet could be a viable option	1	2	3	4	5
5. Follow-up and stabilization after in-person therapy by a psychiatrist, psychologist, or psychotherapist via Internet, email, or phone is feasible	1	2	3	4	5
6. I would definitely recommend online therapy to patients in need of psychiatric, psychological, or psychotherapeutic treatment, if not clinically contraindicated (medical version)<br>For my patients, I would offer online support and propose digital psychiatry interventions via Internet or phone (psychotherapist/psychologist/TRP version)	1	2	3	4	5
7. In addition to face-to-face therapy, it is feasible to combine a psychoeducational, psychosocial, or complementary intervention via Internet or digital psychiatry	1	2	3	4	5
8. Online therapy for mental disorders can only be effective when combined with live contact with the therapist (e.g., via video call, email, or chat)	1	2	3	4	5

1 = Very negatively 2 = Negatively 3 = Neutrally 4 = Positively 5 = Very positively

How do you think your colleagues would judge you if you decided to integrate digital psychiatry interventions into your daily clinical practice?	1	2	3	4	5
How do you think your patients, family, or acquaintances would judge you if you decided to integrate digital psychiatry interventions into your daily clinical practice?	1	2	3	4	5

Digital Readiness (Technology Readiness Scale 2.0./TRI 2.0)

1 = Strongly disagree 2 = Disagree 3 = Neutral 4 = Agree 5 = Strongly agree

1. New technologies contribute to a better quality of life	1	2	3	4	5
2. Technology gives me more freedom of movement	1	2	3	4	5
3. Technology gives people greater control over their daily lives.	1	2	3	4	5
4. Technology makes me more productive in my personal life	1	2	3	4	5
5. Other people ask me for help with new technologies	1	2	3	4	5
6. In general, I am among the first in my circle of friends to acquire new technologies as soon as they are released	1	2	3	4	5
7. I usually understand new tech products and services without needing help from others	1	2	3	4	5
8. I stay up-to-date with the latest technological developments in areas that interest me	1	2	3	4	5
9R. When I get tech support from a high-tech product or service provider, I sometimes feel like I'm taking advantage of someone who knows much more than I do	1	2	3	4	5
10R. Sometimes I think technological systems were not designed to be used by ordinary people	1	2	3	4	5
11R. Technical support hotlines are not helpful because they don't explain things in terms I can understand	1	2	3	4	5
12R. There is no manual for high-tech products or services written in plain language	1	2	3	4	5
13R. People depend too much on technology to do things themselves	1	2	3	4	5
14R. Too much technology distracts people to the point of becoming harmful	1	2	3	4	5
15R. Technology reduces the quality of relationships by limiting personal interactions	1	2	3	4	5
16R. I don't feel comfortable working in a place that can only be accessed online	1	2	3	4	5

Satisfaction and Feasibility

1 = Strongly disagree   2 = Disagree   3 = Neutral   4 = Agree   5 = Strongly agree

1. I was satisfied with my orientation in delivering the digital psychiatry intervention	1	2	3	4	5
2. I was satisfied with the quality of the digital psychiatry service delivered.	1	2	3	4	5
3. The audio quality was acceptable.	1	2	3	4	5
4. The technology (regarding the process that allowed me to deliver the intervention, not the technical issues I may have encountered) distracted me during the therapeutic session	1	2	3	4	5
5. The inability to physically examine my patient definitely compromised the diagnostic process.	1	2	3	4	5
6. I was able to accurately observe symptoms.	1	2	3	4	5
7. I was unable to observe facial expressions and body movements in detail, and this definitely compromised my ability to attune with the patient.	1	2	3	4	5
8. The clinician–patient relationship was negatively affected by the use of digital psychiatry.	1	2	3	4	5
9. I would have preferred to see the patient in person.	1	2	3	4	5
10. Technical difficulties wasted too much time during the process.	1	2	3	4	5
11. My communication with the patient and/or with the patient's caregiver was impaired due to the use of digital psychiatry.	1	2	3	4	5
12. Overall, the system was acceptable and easy to use.	1	2	3	4	5
13. Using digital psychiatry interventions takes more time than face-to-face interventions.	1	2	3	4	5
14. If I had encountered any problems during service delivery, I would have had someone to ask for help.	1	2	3	4	5
15. The digital psychiatry intervention delivered today may have improved my patient's prognosis.	1	2	3	4	5

16. My patient seemed satisfied with the digital psychiatry intervention.	1	2	3	4	5
17. My patient expressed a willingness to use digital psychiatry interventions again.	1	2	3	4	5
18. Overall, I was satisfied with the digital psychiatry intervention I provided.	1	2	3	4	5
19. I would use digital psychiatry interventions again to see my patients.	1	2	3	4	5
20. I would recommend digital psychiatry interventions to my colleagues.	1	2	3	4	5
21. Digital psychiatry improves clinical efficiency.	1	2	3	4	5
22. The digital psychiatry intervention provided today may have made it easier for my patient to access care.	1	2	3	4	5
23. The intervention provided today may have allowed my patient to access services more quickly than with an in-person session.	1	2	3	4	5
24. If the digital psychiatry intervention had not been available today, my patient would have had to travel to receive care.	1	2	3	4	5
25. The digital psychiatry intervention provided today saved time for my patient	1	2	3	4	5
